# Glycine-Binding Site Stimulants of NMDA Receptors Alleviate Extrapyramidal Motor Disorders by Activating the Nigrostriatal Dopaminergic Pathway

**DOI:** 10.3390/ijms18071416

**Published:** 2017-07-03

**Authors:** Saki Shimizu, Shunsaku Sogabe, Ryoto Yanagisako, Akiyoshi Inada, Megumi Yamanaka, Higor A. Iha, Yukihiro Ohno

**Affiliations:** Laboratory of Pharmacology, Osaka University of Pharmaceutical Sciences, 4-20-1 Nasahara, Takatsuki, Osaka 569-1094, Japan; s.shimizu@gly.oups.ac.jp (S.S.); e10611@gap.oups.ac.jp (S.S.); e10817@gap.oups.ac.jp (R.Y.); e10832@gap.oups.ac.jp (A.I.); e10126@gap.oups.ac.jp (M.Y.); k14001@gly.oups.ac.jp (H.A.I.)

**Keywords:** antipsychotics, d-cycloserine, extrapyramidal side effects, glycine-binding site agonists of NMDA receptors, nigrostriatal dopaminergic system, schizophrenia

## Abstract

Dysfunction of the *N*-methyl-d-aspartate (NMDA) receptor has been implicated in the pathogenesis of schizophrenia. Although agonists for the glycine-binding sites of NMDA receptors have potential as new medication for schizophrenia, their modulation of antipsychotic-induced extrapyramidal side effects (EPS) has not yet been clarified. We herein evaluated the effects of glycine-binding site stimulants of NMDA receptors on antipsychotic-induced EPS in mice and rats. d-cycloserine (DCS) and d-serine significantly improved haloperidol (HAL)-induced bradykinesia in mice, whereas glycine showed no effects. Sodium benzoate, a d-amino acid oxidase inhibitor, also attenuated HAL-induced bradykinesia. Improvements in HAL-induced bradykinesia by DCS were antagonized by the NMDA antagonist dizocilpine or nitric oxide synthase inhibitor L-N^G^-Nitro-l-arginine methyl ester. In addition, DCS significantly reduced HAL-induced Fos expression in the dorsolateral striatum without affecting that in the nucleus accumbens. Furthermore, a microinjection of DCS into the substantia nigra pars compacta significantly inhibited HAL-induced EPS concomitant with elevations in dopamine release in the striatum. The present results demonstrated for the first time that stimulating the glycine-binding sites of NMDA receptors alleviates antipsychotic-induced EPS by activating the nigrostriatal dopaminergic pathway, suggesting that glycine-binding site stimulants are beneficial not only for efficacy, but also for side-effect management.

## 1. Introduction

Schizophrenia is a heterogenous disease with diverse psychotic symptoms including positive and negative symptoms, neurocognitive impairments, and mood disturbances [1,2,3,4]. It is well-known that hyperactivity of the meso-limbic dopaminergic system is involved in the pathogenesis of schizophrenia (dopamine hypothesis), and numerous first-generation antipsychotics, which commonly antagonize dopamine D_2_ receptors, have been developed [4,5,6]. These agents effectively improve positive symptoms (e.g., hallucinations, delusion, and excitement) in patients with schizophrenia through D_2_ receptor blockade in the limbic regions (e.g., the nucleus accumbens) [5]. However, they frequently induce extrapyramidal side effects (EPS) by blocking D_2_ receptors in the basal ganglia (e.g., the striatum). In addition, first generation antipsychotics were only effective for positive symptoms, not negative symptoms or cognitive impairments, suggesting that multiple mechanisms (e.g., serotonergic and glutamatergic systems) other than the dopaminergic system are also involved in the generation of schizophrenia symptoms [1,7,8,9]. 

*N*-methyl-d-aspartate (NMDA) receptors are heteromeric tetramer proteins composed of GluN1 and GluN2 subunits containing d-serine/glycine- and glutamate-binding sites, respectively [10,11]. Besides d-serine/glycine- and glutamate-binding sites, they also possess several regulatory sites sensitive to polyamines, Zn^2+^, protons, and glutathione [10,12]. NMDA receptors are involved in the etiology and treatment of various neuropsychiatric disorders (e.g., schizophrenia, depression, Alzheimer’s disease, and ischemic stroke) [13,14,15,16,17,18]. Previous studies proposed that the dysfunction of NMDA receptors is involved in the pathogenesis of schizophrenia (the glutamate hypothesis in schizophrenia) [13,14,15]. This hypothesis was derived from agents including phencyclidine (PCP) and dizocilpine (MK-801), which block the function of NMDA receptors and cause psychosis in humans, inducing schizophrenia-like symptoms [13,19,20,21]. It is also supported by previous findings showing decreased glutamate levels in the cerebrospinal fluid [22] and the down-regulation of brain NMDA receptor expression in patients with schizophrenia [23]. Furthermore, based on the glutamate hypothesis in schizophrenia, several agents (e.g., d-cycloserine (DCS), d-serine, and sodium benzoate), which stimulate NMDA receptor functions, are expected to have potential as new medication for schizophrenia [14,15,24,25]. However, their actions regarding the induction and/or modulation of EPS are unknown and remain to be clarified. 

In the present study, we performed behavioral and immunohistochemical studies in mice and rats to evaluate the effects of the glycine-binding site stimulants of NMDA receptors on antipsychotic-induced EPS (i.e., bradykinesia and catalepsy) and elucidate their action mechanisms.

## 2. Results

### 2.1. Effects of Glycine-Site Stimulants of N-Methyl-d-aspartate (NMDA) Receptors on Haloperidol-Induced Bradykinesia

**Figure 1:**
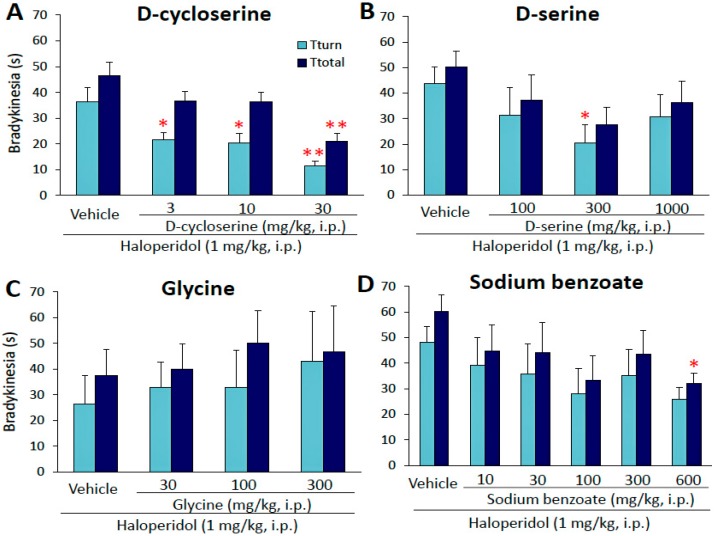
Effects of glycine-site agonists of *N*-methyl-d-aspartate (NMDA) receptors and the d-amino acid oxidase inhibitor on haloperidol (HAL)-induced bradykinesia. (**A**–**D**) Glycine-site agonists of NMDA receptors, d-cycloserine (3–30 mg/kg, i.p.), d-serine (100–1000 mg/kg, i.p.), and glycine (30–300 mg/kg, i.p.), and the d-amino acid oxidase inhibitor, sodium benzoate (10–600 mg/kg, i.p.), were administered to animals 15 min before the HAL injection. The pole test was performed 30 min after the HAL injection. Each column represents the mean ± S.E.M. of 5–13 mice. These data were analyzed using the Kruskal−Wallis and Steel−Dwass tests. * *p* < 0.05, ** *p* < 0.01, significantly different from the value with HAL alone.

We examined the effects of the glycine-site stimulants of NMDA receptors on haloperidol (HAL)-induced bradykinesia using the mouse pole test. The glycine-site agonist of NMDA receptors, DCS (3–30 mg/kg, i.p.), significantly improved HAL (1 mg/kg, i.p.)-induced bradykinesia in a dose-dependent manner (*T*_turn_: *F*(3,44) = 7.8073, *p* = 0.0003, *T*_total_: *F*(3,44) = 6.7772, *p* = 0.0007) (Figure 1A). d-serine (100–1000 mg/kg, i.p.) significantly attenuated HAL-induced bradykinesia (*T*_turn_: *X*^2^ *=* 8.4239, df = 3, *p* = 0.0380), whereas glycine (30–300 mg/kg, i.p.) showed no effects (Figure 1B,C). In addition, the d-amino acid oxidase inhibitor, sodium benzoate (600 mg/kg, i.p.), also significantly reduced HAL-induced bradykinesia (*T*_total_: *X*^2^ *=* 8.7330, df = 5, *p* = 0.0481) (Figure 1D).

**Figure 2:**
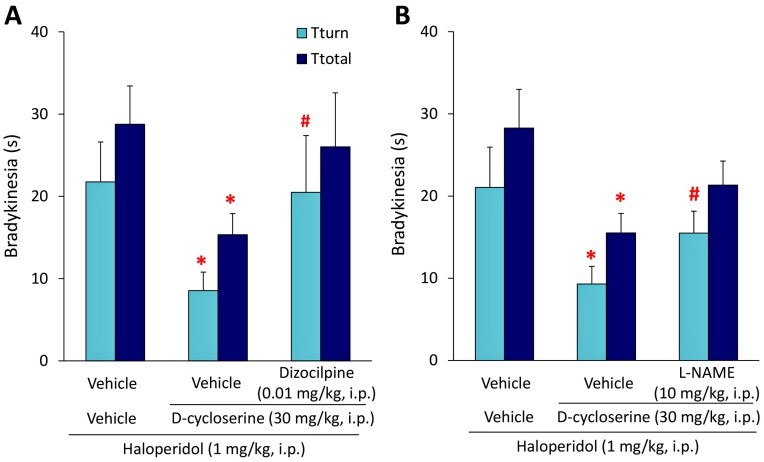
Effects of dizocilpine or L-N^G^-Nitro-l-arginine methyl ester (L-NAME) on the ameliorative action of d-cycloserine (DCS) against haloperidol (HAL)-induced bradykinesia. (**A**,**B**) Mice received dizocilpine (0.01 mg/kg, i.p.), L-NAME (10 mg/kg, i.p.), or vehicle simultaneously with DCS (30 mg/kg, i.p.) or vehicle 15 min before the HAL injection (1 mg/kg, i.p.). The pole test was performed 30 min after the HAL injection. Each column represents the mean ± S.E.M. of 12–23 mice. These data were analyzed using the Mann-Whitney *U*-test. * *p* < 0.05: Significantly different from the value with HAL alone. ^#^ *p* < 0.05, significantly different from the value with HAL + DCS.

We subsequently examined the effects of the NMDA receptor antagonist dizocilpine and nitric oxide synthase (NOS) inhibitor L-N^G^-Nitro-l-arginine methyl ester (L-NAME) on the antibradykinetic action of DCS. Dizocilpine (0.01 mg/kg, i.p.), L-NAME (10 mg/kg, i.p.), or vehicle was simultaneously injected with DCS (30 mg/kg, i.p.) 15 min before the HAL injection (1 mg/kg, i.p.). Under these conditions, the improvement of HAL-induced bradykinesia by DCS was significantly antagonized by dizocilpine (0.01 mg/kg, i.p.) (*T*_turn_: *U* = 47, *p* = 0.0066) (Figure 2). Similarly, L-NAME (10 mg/kg, i.p.) also significantly antagonized improvements by DCS (*T*_turn_: *U* = 144.5, *p* = 0.0137) (Figure 2).

### 2.2. Effects of d-Cycloserine on Haloperidol-Induced Fos Expression

It is well-known that HAL evokes Fos protein expression, a biological marker of neural excitation [26], in the forebrain (e.g., striatum and nucleus accumbens) via dopamine D_2_ receptor blockade [27,28]. Therefore, we examined the effects of an anti-bradykinetic dose of DCS on HAL-induced Fos expression in the dorsolateral striatum (dlST) and shell region of the nucleus accumbens (AcS). 

**Figure 3:**
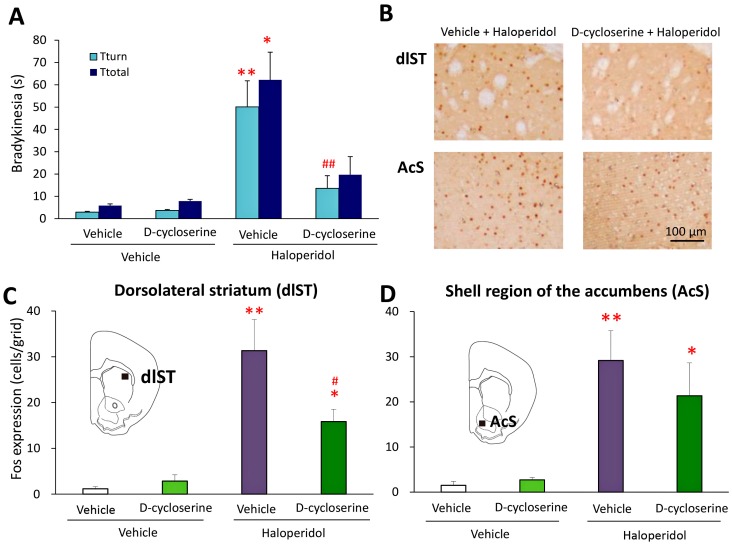
(**A**) Effects of d-cycloserine (DCS) on haloperidol (HAL)-induced Fos expression in the dorsolateral striatum (dlST) and accumbens shell (AcS). (**A**) DCS (30 mg/kg, i.p.) or vehicle was administered to animals 15 min before the HAL injection, which was followed by the pole test 30 min later; (**B**) Photographs illustrating Fos-IR-positive cells in the dlST and AcS (**left** panel: vehicle + HAL (1 mg/kg, i.p.)-treated mice, **right** panel: DCS (30 mg/kg, i.p.) + HAL (1 mg/kg, i.p.)-treated mice). Scale bar: 100 μm; (**C**,**D**) Effects of DCS (30 mg/kg, i.p.) on HAL (1 mg/kg, i.p.)-induced Fos expression in the dlST (**C**) and AcS (**D**). The brain was removed from animals 2 h after the HAL injection. Each column represents the mean ± S.E.M. of 6–7 mice. These data were analyzed using the Kruskal−Wallis and Steel−Dwass tests (behavioral test) or one-way ANOVA and Tukey’s test (Fos analysis). * *p* < 0.05, ** *p* < 0.01, significantly different from the value for vehicle + vehicle. ^#^ *p* < 0.05, ^##^ *p* < 0.01, significantly different from the value for vehicle + HAL.

As shown in Figure 3A, we confirmed that HAL (1 mg/kg, i.p.) markedly increased *T*_turn_ and T_total_ values, which were significantly reversed by DCS (30 mg/kg, i.p.). Brain samples were then obtained from these animals 2 h after the HAL injection and subjected to Fos immunohistochemistry. Under these conditions, control (vehicle + vehicle) and DCS (vehicle + DCS) animals showed negligible Fos expression in the dlST and AcS. The number of Fos immunoreactivity (IR)-positive cells was markedly increased by HAL (vehicle + HAL, dlST: *F*(3,21) = 14.9794, *p* = 0.0001, AcS: *F*(3,21) = 8.3832, *p* = 0.0030) (Figure 3B–D). However, HAL-induced Fos expression was significantly inhibited by DCS in the dlST (*p* = 0.0324). The number of Fos-IR-positive cells in the dlST was approximately 31 cells/grid with vehicle + HAL; however, this value was reduced to approximately 16 cells/grid by the combined treatment with DCS (Figure 3B,C). Interestingly, DCS did not significantly affect HAL-induced Fos expression in the AcS (Figure 3B,D).

### 2.3. Microinjection and In Vivo Microdialysis Studies with d-Cycloserine

**Figure 4:**
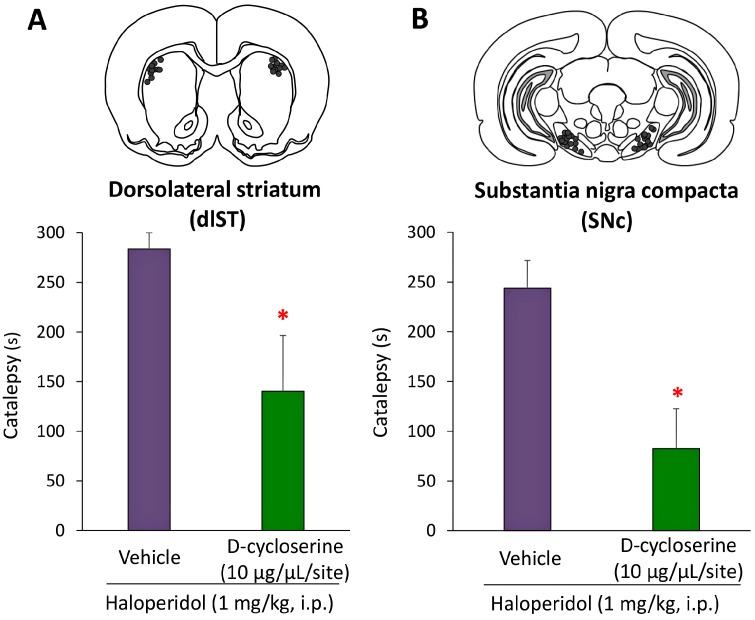
Effects of intranigral and intrastriatal microinjections of d-cycloserine (DCS) on haloperidol (HAL)-induced catalepsy in rats. (**A**,**B**) The effects of DCS (10 µg/1 µL/side) microinjected into the substantia nigra compacta (SNc) or dorsolateral striatum (dlST) against HAL-induced catalepsy were examined. Each dose of HAL was administered 15 min after each DCS microinjection and, 30 min later (45 min after the DCS microinjection), the catalepsy time was measured. Schematic drawings of a rat brain section illustrating DCS or vehicle injection sites (filled circles) in the SNc (**A**) or dlST (**B**) are shown at the top. Each column represents the mean ± S.E.M. of 6–11 rats. These data were analyzed by the Mann-Whitney *U*-test. * *p* < 0.05, significantly different from the control value with vehicle + HAL.

In order to investigate the action sites of DCS, we conducted microinjection studies using rats. DCS (10 μg/site) or vehicle was microinjected into the bilateral substantia nigra pars compacta (SNc) or dlST 15 min after the HAL (1 mg/kg, i.p.) treatment. HAL-induced EPS was evaluated by the catalepsy test 30 min after the HAL injection. Under these conditions, microinjections of DCS into the SNc or dlST significantly attenuated HAL-induced catalepsy (Figure 4). The catalepsy time with HAL was significantly reduced from 243.6 ± 28.0 to 82.7 ± 39.9 (SNc: *U* = 17, *p* = 0.0123) and 283.8 ± 16.2 to 140.5 ± 56.0 (dlST: *U* = 7, *p* = 0.0495) by the microinjection of DCS into the SNc and dlST, respectively. 

**Figure 5:**
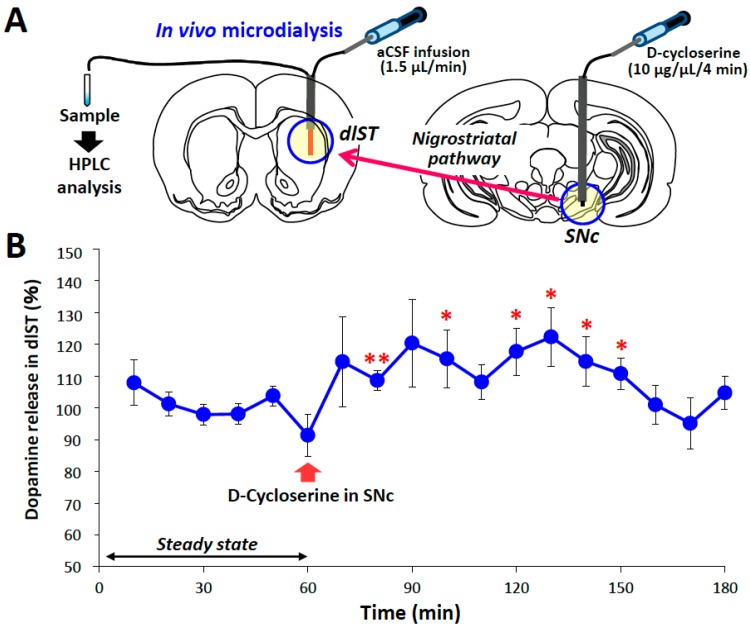
Effects of an intranigral microinjection of d-cycloserine (DCS) on dopamine release in the rat striatum. (**A**) Schematic drawing illustrating the microinjection site of DCS in the substantia nigra pars compacta (SNc) and microdialysis site in the dorsolateral striatum (dlST); (**B**) Extracellular levels of dopamine were analyzed in 10-min dialysate samples. Data were normalized to the mean value of the first six 10-min samples (basal value). Each column shows the mean ± S.E.M. of 6 rats. These data were analyzed by the Student’s *t*-test. * *p <* 0.05, ** *p <* 0.01, significantly different from the basal value.

We further conducted in vivo microdialysis studies to evaluate extracellular dopamine release in the dlST following the microinjection of DCS (10 μg/site) into the ipsilateral SNc (Figure 5). The microinjection of DCS into the SNc significantly enhanced dopamine release in the ipsilateral dlST. Extracellular dopamine levels were significantly elevated by approximately 25% by the microinjection of 10 μg DCS into the SNc (100 min: *T* = 2.2701, df = 10, *p* = 0.0466).

## 3. Discussion

The present study demonstrated that the glycine-binding site agonists of NMDA receptors, DCS and d-serine, significantly alleviated HAL-induced bradykinesia with relative potencies in the order of DCS > d-serine. Sodium benzoate, an inhibitor of the d-amino acid oxidase catalyzing the oxidative metabolism of d-serine [29,30], also alleviated HAL-induced bradykinesia. Thus, sodium benzoate appears to alleviate HAL-induced bradykinesia by increasing extracellular d-serine levels 

Although d-serine-independent mechanisms (e.g., NMDA receptor redox-site intervention) are also proposed [31,32]. Among the glycine-binding site stimulants tested, glycine was inactive. The lack of efficacy with glycine may have been due to its poor penetration into the brain or an insufficient dosage. However, it was not possible to test higher doses of glycine because of its acute toxicity (occasional death at 1000 mg/kg).

Antibradykinetic doses of the glycine-binding site stimulants of NMDA receptors in the pole test were 3–30 mg/kg (i.p.) for DCS, 300 mg/kg (i.p.) for d-serine, and 600 mg/kg (i.p.) for sodium benzoate. These doses were similar to those producing efficacy in animal models of schizophrenia with NMDA antagonists (e.g., phencyclidine and dizocilpine), namely, 10–30 mg/kg (s.c.) for d-cycloserine, 600 mg/kg (i.p.) for d-serine, and 300–1000 mg/kg (p.o.) for sodium benzoate [31,32,33]. Therefore, the glycine-binding site stimulants of NMDA receptors are expected to reduce EPS associated with antipsychotic treatments in clinical settings.

It has been well-documented that antipsychotics elevate the regional expression of the Fos protein, a biological marker of neural activation, both in the nucleus accumbens and striatum by blocking D_2_ receptors [34]. Furthermore, D_2_ receptor-mediated Fos expression in the nucleus accumbens and striatum are considered to reflect the antipsychotic action and EPS liability of antipsychotics, respectively [27,35,36,37,38]. Second-generation antipsychotics (atypical antipsychotics) with fewer EPS commonly lead to reduced Fos expression in the striatum [27,28,39,40]. In the present study, we showed that DCS significantly reduced Fos expression in the dlST. This evidence further supports DCS counteracting striatal D_2_ receptor blockade by HAL to attenuate the induction of EPS. The effects of DCS on Fos expression were region-specific and did not significantly alter HAL-induced Fos expression in the AcS. These results suggest that a combination of the glycine-binding site stimulants of NMDA receptors with antipsychotics preferentially attenuates EPS (D_2_ blocking action in the striatum) without interfering with the therapeutic action of antipsychotics.

In order to elucidate the action mechanisms of DCS, we first examined the effects of dizocilpine (NMDA antagonist) and confirmed that it antagonized improvements in HAL-induced bradykinesia by DCS, indicating that DCS alleviated EPS by activating NMDA receptors. Since NMDA receptor-mediated neurotransmission is known to be mediated by NO synthesis, we also examined the effects of the NOS inhibitor L-NAME and showed that it antagonized DCS-induced improvements in HAL-induced bradykinesia. Therefore, NMDA receptor-mediated NO production appears to be involved in the antiparkinsonian action of DCS. In addition, we performed microinjection studies in combination with in vivo microdialysis measurements of dopamine release in the striatum. Our results demonstrated that microinjections of DCS (10 µg/site/4 min) into the SNc or dlST significantly improved HAL-induced catalepsy in rats, indicating that SNc and dlST are both, at least partly, involved in the anti-cataleptic action of DCS. In vivo microdialysis results also revealed that DCS locally injected into the SNc significantly enhanced dopamine release in the dlST. These results suggest that the stimulation of glycine-binding sites by DCS in the SNc activates the nigrostriatal dopamine pathway, which reduces EPS by elevating striatal dopamine increases. On the other hand, the mechanisms of action of DSC in the striatum currently remain unknown. Since the suppression of striatal medium spiny neurons leads to the amelioration of extrapyramidal disorders [41], the activation of NMDA receptors by DCS may inhibit medium spiny neurons via inhibitory GABAergic interneurons. Further studies are needed in order to elucidate the action mechanisms of DSC in the striatum in more detail.

In conclusion, we herein investigated the actions of glycine-binding site stimulants of NMDA receptors in the modulation of antipsychotic-induced EPS and showed that DCS, d-serine, and sodium benzoate significantly attenuated HAL-induced EPS. The anti-EPS action of DCS was antagonized by dizocilpine and L-NAME, suggesting that the activation of NMDA receptors and subsequent induction of NO were involved in the amelioration of EPS by DCS. In addition, DCS counteracted HAL-induced Fos expression in the striatum, but not in the nucleus accumbens, suggesting that the glycine-binding site stimulants of NMDA receptors preferentially attenuate the D_2_ blocking action of antipsychotics in the striatum compared with that in the striatum. Furthermore, the microinjection of DCS into the SNc effectively improved HAL-induced EPS concomitant with elevations in dopamine release in the striatum. The present results suggest that the stimulation of glycine-binding sites of NMDA receptors alleviates antipsychotic-induced EPS by activating nigrostriatal dopamine neurons. Based on the glutamate hypothesis, the glycine-binding site stimulants of NMDA receptors are expected to become a new medication for schizophrenia [42]. Clinical studies showed that several agents including DCS and sodium benzoate improved negative symptoms and/or cognitive impairment in patients with schizophrenia [43,44,45,46]. The present results suggest that glycine-binding site agonists are beneficial not only for efficacy, but also side-effect management in the treatment of schizophrenia. However, due to the limitations associated with animal experiments, further clinical studies are needed in order to validate the EPS liability and/or antiparkinsonian effects of glycine-binding site stimulants of NMDA receptors.

## 4. Materials and Methods 

### 4.1. Animals

Male ddY mice and SD rats (Japan SLC, Shizuoka, Japan) at 8–10 weeks of age were used. Animals were kept in air-conditioned rooms (24 ± 2 °C and 50 ± 10% relative humidity) under a 12-h light/dark cycle (light on: 8:00–20:00) and allowed free access to food and water. Animal care methods complied with the Guide for the Care and Use of Laboratory Animals of the Ministry of Education, Science, Sports and Culture of Japan, and experimental protocols were approved by the Experimental Animal Research Committee at Osaka University of Pharmaceutical Sciences (#17, 30 March 2015).

### 4.2. Evaluation of Bradykinesia

The pole test was performed as described previously [47]. Mice were placed at the top (head-upward) of a pole (diameter: 8 mm and height: 45 cm). The time for the animal to rotate downward (*T*_turn_) and descend to the floor (*T*_total_) was then measured with a cut-off time of 90 s. Only mice that showed *T*_turn_ < 8 s and *T*_total_ < 18 s in the pre-test trial (typically performed 2 h before the test trial) were used. 

The glycine-site stimulants of NMDA receptors, DCS (3–30 mg/kg, i.p.), d-serine (100–1000 mg/kg, i.p.), and glycine (30–300 mg/kg, i.p.), and the d-amino acid oxidase inhibitor, sodium benzoate (10–600 mg/kg, i.p.) were administered to animals 15 min before the HAL injection, and the pole test was performed 30 min later. In experiments using dizocilpine or l-NAME, mice first received dizocilpine (0.01 mg/kg, i.p.), l-NAME (10 mg/kg, i.p.), or vehicle simultaneously injected with DCS or vehicle 15 min before the HAL injection (1 mg/kg, i.p.). The pole test was performed 30 min after the HAL injection.

### 4.3. Analysis of Fos Protein Expression

Regarding Fos immunohistochemical staining, brain samples were obtained from mice 120 min after the HAL injection. Under pentobarbital (80 mg/kg, i.p.) anesthesia, all mice were transcardially perfused with ice-cold phosphate-buffered saline (PBS), which was followed by 4% formaldehyde perfusion. Brains were removed from the skull and stored in fresh fixative for at least 24 h. 

Fos immunohistochemical staining was performed using previously reported methods [48,49]. Coronal sections (thickness: 30 μm) were cut from the brain using a Microslicer (DSK-3000, Dosaka, Kyoto, Japan). Slices were incubated for 2 h with 2% normal rabbit serum, and with goat c-Fos antiserum (Santa Cruz Biotechnology Inc., Santa Cruz, CA, USA) for an additional 18–36 h. Sections were then incubated with a biotinylated rabbit anti-goat IgG secondary antibody (Vector Laboratories, Burlingame, CA, USA) for 2 h. After a 30 min incubation with 0.3% hydrogen peroxide for 30 min to inactivate endogenous peroxidase, sections were incubated for 2 h with an avidin–biotinylated horseradish peroxidase complex (Vectastain ABC Kit, Vector Laboratories, Burlingame, CA, USA). Fos-IR was visualized using the diaminobenzidine–nickel staining method and quantified by counting the number of Fos-IR positive nuclei in dlST and AcS. 

### 4.4. Microinjection Study

DCS was microinjected into the SNc (−6.0 mm anterior to the bregma, ±2.2 mm lateral to the midline, 6.2 mm inferior to the brain surface) or dlST (+1.0 mm anterior to the bregma, ±1.0 mm lateral to the midline, 3.5 mm inferior to the brain surface) [50] in rats, according to a previously reported method [51]. Under pentobarbital (80 mg/kg, i.p.) anesthesia, male SD rats were fixed in a stereotaxic frame (Narishige, SR-6, Tokyo, Japan). Stainless steel-guide cannulae were then inserted into a position 1 mm above the bilateral SNc or dlST and fixed to the skull with dental cement. After a recovery period (ca. 1 week), animals were subjected to microinjection experiments. 

On the day of the experiment, injection cannulae were inserted into the SNc or dlST through the guide cannulae. Under freely-moving conditions, DCS (10 μg/1 μL/site) was injected into the SNc or dlST at a flow rate of 0.25 μL/min for 4 min using a microinjection pump (KDS220; Kd Scientific Inc., Holliston, MA, USA). Control animals were given the same volume of vehicle alone. Fifteen min after the DCS microinjection, animals were injected with HAL (1 mg/kg, i.p.) and, 30 min later, subjected to the catalepsy test in order to evaluate the induction of EPS [51]. When the same animals were treated with a different drug (or vehicle) solution, the microinjection study was performed after a drug withdrawal period of at least 4 days. After experiments, the brain was removed from animals under pentobarbital (80 mg/kg, i.p.) anesthesia, and the position of each injection site was checked. 

### 4.5. In Vivo Microdialysis Study

Under pentobarbital (40 mg/kg, i.p.) anesthesia, male SD rats were fixed in a stereotaxic instrument (Narishige, SR-6, Tokyo, Japan). A guide cannula (diameter: 1 mm) was inserted into a position 1 or 2 mm above the unilateral SNc or dlST and fixed to the skull using dental cement. After a recovery period (ca. 1 week), animals were subjected to in vivo microdialysis experiments. Briefly, a dialysis probe (Eicom, A-I-6-02, Kyoto, Japan) was inserted into the dlST through a guide cannula and artificial cerebrospinal fluid (aCSF), containing NaCl 140 mM, KCl 2.4 mM, MgCl_2_ 1.0 mM, CaCl_2_ 1.2 mM, and NaHCO_3_ 5.0 mM, was perfused at a flow rate of 1.5 μL/min using a microperfusion pump (Eicom, ESP-32, Kyoto, Japan). Under freely-moving conditions, animals were given DCS: 10 μg/1 μL/site was slowly injected into the SNc at flow rate of 0.25 μL/min for 4 min using a microinfusion pump (KDS220; Kd Scientific Inc., Holliston, MA, USA). Dialysate samples were collected into a microtube every 10 min (15 μL/sample), and analyzed for dopamine levels using an HPLC-ECD system. The mobile phase consisted of 0.1 M acetic acid-citric acid buffer, 190 mg/L 1-octanesulfonic acid sodium, 5 mg/L EDTA 2 Na, pH 3.5, with 16% methanol pumped at a flow rate of 230 μL/min. All data were analyzed using eDAQ Power Chrom (eDAQ Pty Ltd., Denistone East, NSW, Australia). Extracellular dopamine levels were expressed as a percentage of the basal control level (steady state), which was the mean of six points before the application of DCS, in each animal. 

After experiments, animals were deeply anesthetized with pentobarbital (80 mg/kg, i.p.) and the brain was removed from the skull. Coronal sections (thickness of 100 µm) were prepared from each brain using a microslicer (DSK, Kyoto, Japan) and the position of each injection site was checked.

### 4.6. Drugs

HAL, DCS, d-serine, glycine, sodium benzoate, dizocilpine, L-NAME, and CNQX were purchased from Sigma-Aldrich (St. Louis, MO, USA). The Vectastain ABC kit and DAB substrate were purchased from Vector Laboratories (Burlingame, CA, USA). All other reagents were obtained from commercial sources. HAL was dissolved in 1% lactate solution and then diluted with physiological saline. Other agents were dissolved in physiological saline. All drugs were injected intraperitoneally in a volume of 5 mL/kg into mice or 1 mL/kg into rats. 

### 4.7. Statistical Analysis

Data are expressed as the mean ± S.E.M. The significance of differences among multiple groups was assessed by a one-way ANOVA followed by Tukey’s test or Kruskal−Wallis test (nonparametric one-way ANOVA) followed by the Steel−Dwass post-hoc test. Comparisons between only two groups were performed by the non-parametric Mann-Whitney *U*-test or parametric Student’s *t*-test. A *p*-value of less than 0.05 was considered significant.

## Abbreviations

AcS	Shell region of the nucleus accumbens
DCS	d-Cycloserine
dlST	Dorsolateral striatum
EPS	Extrapyramidal side effects
HAL	Haloperidol
IR	Immunoreactivity
L-NAME	L-N^G^-Nitro-l-arginine methyl ester
NMDA	*N*-Methyl-d-aspartate
NOS	Nitric oxide synthase
SNc	Substantia nigra pars compacta
